# Abdominal distension resulting from hematocolpos in children: A case report

**DOI:** 10.1016/j.ijscr.2024.110414

**Published:** 2024-10-04

**Authors:** Aicha Chebil, Haifa Bouchahda, Sabrine Nouiji, Hela Dahmeni, Mohamed Ali Chaouch, Khouloud Marzouk

**Affiliations:** a Gynecology Department of Tahar Sfar University Hospital, Mahdia, Tunisia; b Department of Visceral and Digestive Surgery, Monastir University Hospital, Monastir, Tunisia

**Keywords:** Pediatric abdominal pain, Amenorrhea, Hematocolpos, Imperforate hymen, Case report

## Abstract

**Introduction:**

Hymeneal imperforation is a rare genital malformation often discovered during abdominopelvic emergencies. Hematocolpos typically presents with pelvic pain, a palpable mass, and primary amenorrhoea. The diagnosis is confirmed by clinical evaluation and imaging studies such as ultrasound.

**Case presentation:**

A 13-year-old girl presented a four-week history of abdominal distension and pain, which worsened over time, along with constipation, but without vomiting or fever. Examination revealed stable vital signs and abdominal guarding. A gynecological exam showed an imperforate hymen. Ultrasound identified a hypoechoic fluid collection in the retrovesical area. Hematocolpos was diagnosed and surgical intervention involved opening the hymen and releasing 800 cc of blood. The patient remained stable after the operation and was discharged painless after two days.

**Discussion:**

Imperforate hymen, resulting from incomplete resorption of the hymeneal membrane during embryonic development, is a common cause of hematocolpos. Symptoms often manifest in menarche, with cyclical pelvic pain and primary amenorrhoea. The diagnosis is based on physical examination and imaging, while early intervention prevents complications such as endometriosis and infertility. Surgical treatment varies from hymenotomy to more complex reconstructive procedures based on the underlying cause.

**Conclusions:**

Although rare, imperforate hymen is the most prevalent congenital anomaly of the vagina, often remaining asymptomatic until menarche. Accurate diagnosis and timely surgical intervention are essential to avoid severe complications. This case highlights the importance of comprehensive clinical evaluation and appropriate imaging in the management of hematocolpos.

## Highlights

• It highlights the importance of considering imperforate hymen in differential diagnoses for adolescent girls with similar presentations to ensure timely and appropriate management
• It highlights the difficulty in diagnosing hematocolpos due to its rare occurrence and nonspecific symptoms.
• It highlights the critical need for early recognition and treatment of imperforate hymen to prevent severe complications such as infection, endometriosis, and infertility.

## 1 Introduction and importance

Hymeneal imperforation is a rare genital malformation often discovered during abdominopelvic emergencies, estimated to be between 0.05 % and 0.1 % [1]. The diagnosis of hematocolpos can be established through a combination of clinical evaluation and imaging studies. The clinical presentation typically includes pelvic pain, a palpable mass, and primary amenorrhoea. Imaging modalities, such as ultrasound, can confirm the diagnosis by visualising the dilated vagina filled with blood [2]. This case report, presented according to the SCARE guidelines [3], aimed to highlight the main characteristics of hematocolpos in children by showing a typical clinical case.

## 2 Case presentation

**Fig. 1:**
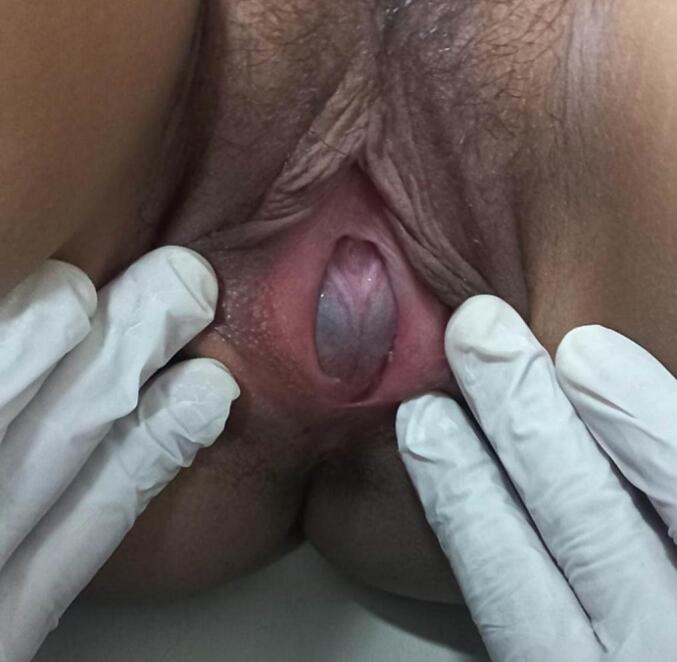
Obstruction of the vaginal orifice by a thin, bluish, and bulging membrane (hymen) in the presence of secondary sexual characteristics.

**Fig. 2:**
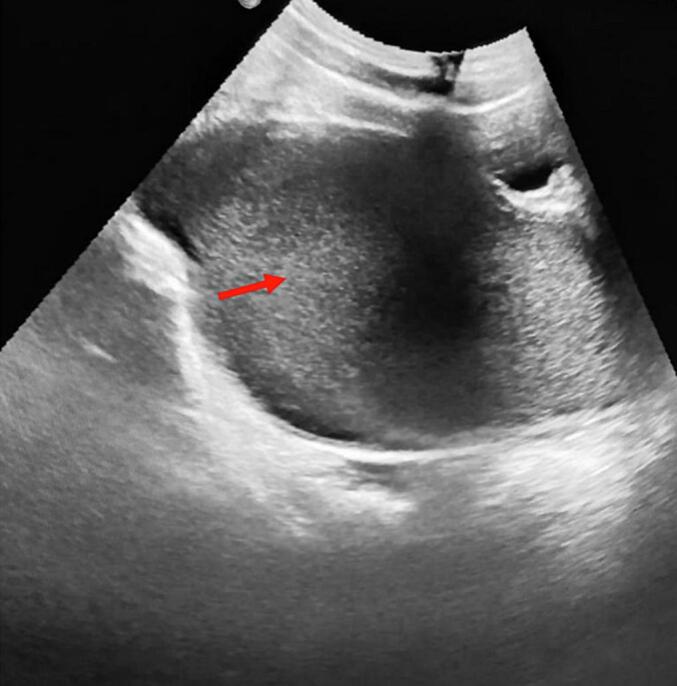
Surpubic ultrasound view of retro-vesical, median, hypoechoic fluid containing a fine echogenic spot (red arrow). (For interpretation of the references to colour in this figure legend, the reader is referred to the web version of this article.)

**Fig. 3:**
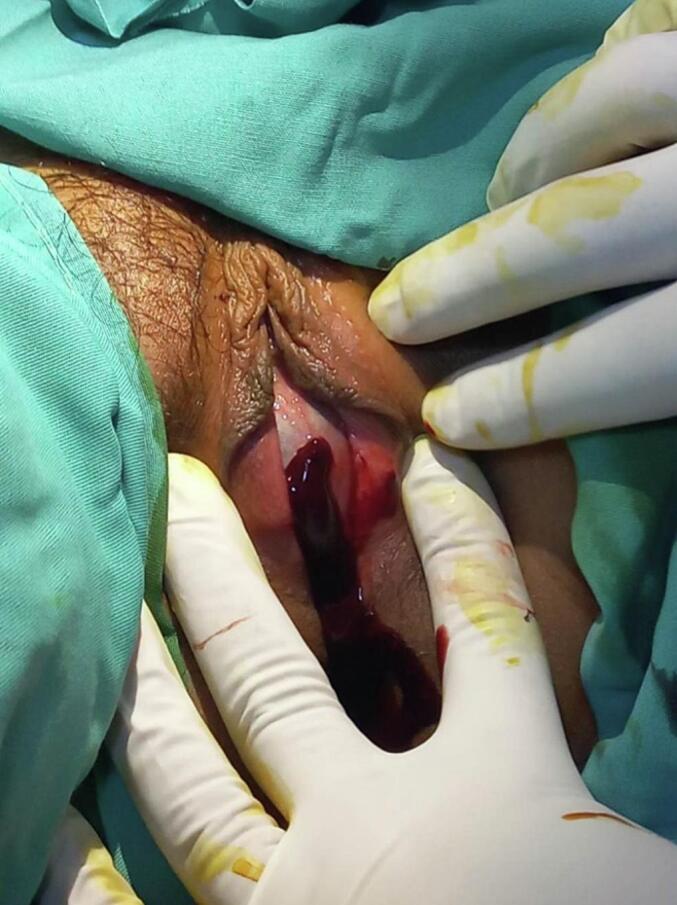
The vaginal membrane was opened using X-shaped incisions, resulting in a significant discharge of 800 cc of blood.

A healthy 13-year-old girl presented to a pediatric emergency department with a four-week history of abdominal distention and pain evaluated at 5 per 10 according to the numerical rating scale that had recently worsened. She also complains of constipation without associated vomiting or fever. The medical history was unremarkable. She did not have any familiar and gynecological history and had never settled. On examination, vital signs showed regular blood pressure of 110/70 mm / Hg, regular pulse of 90 (bpm), regular breathing of 19 per minute, proper capillary filling, temperature of 37.5 °C, good air entry to two lungs and regular heartbeats. She presented total abdominal guarding. There were no rashes on her limbs or other areas of her body, and neurologically there were no discernible marks. A gynecological examination showed obstruction of the vaginal orifice by a thin, bluish, and bulging membrane (hymen) in the presence of secondary sexual characteristics (Fig. 1). Suprapubic ultrasound shows a retrovesical, median, hypoechoic fluid that contains a fine echogenic spot. This collection is supported by the small uterine cavity (Fig. 2). Hematocolpos was diagnosed and the vaginal membrane was opened using X-shaped incisions, resulting in a significant discharge of 800 cc of blood under local anaesthesia (Fig. 3). The girl remained hemodynamically stable with no ongoing bleeding and was discharged in stable and pain-free condition after two days of observation. The patient was seen 30 days in the outpatient clinic and the next menstrual period was uneventful.

## 3 Case discussion

Imperforate hymen is a clinical condition whose aetiology remains uncertain, but it is frequently implicated as the leading cause of hydrometrocolpos. It arises from incomplete resorption of the hymeneal membrane during embryonic development and its failure to rupture during the perinatal period, with irregular maturation [1]. Although familial occurrences have been documented, isolated cases generally lack identifiable mutations or genetic markers [2]. The evaluation of the hymen at birth is crucial, as newborns can have a prominent hymeneal membrane due to mucocolpos. Hematocolpos, on the other hand, is characterised by the accumulation of menstrual blood in the vagina, resulting from various factors that obstruct menstrual blood flow. This condition commonly arises from the combination of menstruation with an imperforate hymen, where the hymen completely covers the vaginal opening, preventing the flow of menstrual blood. Less frequently, hematocolpos may be attributed to uterovaginal malformations, such as complete vaginal diaphragm, partial vaginal atresia, uterus didelphys, or cervical atresia [4]. Furthermore, it should be noted that hematocolpos may be associated with a rare syndrome known as OHVIRA or Herlyn-Werner-Wunderlich syndrome, which requires specialised management [5]. In our region, hematocolpos cases are often diagnosed late due to limited access to health facilities and a lack of awareness of congenital gynecologic anomalies. This contributes to delayed treatment and increases the risk of complications such as endometriosis and infertility. Addressing these conditions in a local context highlights the importance of early detection through community education and improved clinical resources. Furthermore, conditions such as imperforate hymen and other benign gynecologic anomalies such as uterine malformations or pelvic masses are often overlooked until symptoms worsen, particularly in rural areas. Local medical practices and cultural factors also play a role in delayed presentations, as families may hesitate to seek medical advice for young girls with gynecologic problems. Further studies focused on these factors could lead to better preventive strategies and management tailored to local needs. The diagnosis of hematocolpos can be delayed, but early diagnosis is crucial to prevent complications such as endometriosis, infection, and infertility [4,6]. Symptoms of hematocolpos are generally present in puberty during the first menstruation with cyclical pelvic pain, primary amenorrhoea or pelvic mass, palpable mass, and vaginal bleeding or discharge [4,7] and, although urinary difficulty is rare, the appearance of urinary retention [8]. Hematocolpos is rarely diagnosed during the neonatal period. Typically, its diagnosis involves a combination of physical examination, imaging techniques such as ultrasound or MRI, and occasionally genetic tests [5]. Treatment for hematocolpos involves surgical intervention to relieve obstruction and drain the accumulated menstrual blood. The specific surgical approach depends on the underlying cause of the hematocolpos. For example, in cases of imperforate hymen, a simple hymenectomy may be sufficient, while more complex cases may require reconstructive surgery [4]. Alternative approaches to managing hematocolpos, in addition to the hymenectomy mentioned in the article, include minimally invasive techniques such as needle aspiration or small incisions to drain the blood, which may be used in milder cases. Additionally, hormonal therapy can be considered to temporarily suppress menstruation until the patient is ready for surgery, although this is less commonly used.

## 4 Conclusions

The most prevalent congenital anomaly of the vagina is imperforate hymen, although it is relatively rare and may be related to other urogenital malformations. Typically, it remains asymptomatic until menarche, when the accumulation of blood in the internal genitalia becomes evident. The diagnosis typically results from a comprehensive patient history and examination, combined with imaging techniques for confirmation while also excluding associated pathologies. A clinical example of a teenage patient with abdominal mass resulting from hematocolpos due to an imperforate hymen is presented.

## Patient consent

Written informed consent was obtained from the patient to publish this case report and accompanying images. On request, a copy of the written consent is available for review by the Editor-in-Chief of this journal.

## Ethical approval

Ethical approval is exempt/waived at our institution for all the case reports.

## Funding

No funding.

## Author contribution

All the authors participated in the manuscript and validated the final version of the manuscript.

## Guarantor

Mohamed Ali Chaouch.

## Research registration number

Not applicable.

## Declaration of competing interest

The authors declare no conflict of interest.
